# Hidden musculoskeletal involvement in inflammatory bowel disease: a multicenter ultrasound study

**DOI:** 10.1186/s12891-016-0932-z

**Published:** 2016-02-16

**Authors:** João Rovisco, Cátia Duarte, Alberto Batticcioto, Piercarlo Sarzi-Puttini, Antonella Dragresshi, Francisco Portela, Marwin Gutierrez

**Affiliations:** Rheumatology Department, Centro Hospitalar e Universitário de Coimbra, Praceta Prof Mota Pinto, 3000-075 Coimbra, Portugal; Rheumatology Unit, L. Sacco University Hospital, Milan, Italy; Clinica Reumatologica, Università Politecnica delle Marche, Jesi, Ancona, Italy; Gastroenterology Department, Centro Hospitalar e Universitário de Coimbra, Coimbra, Portugal; Division of Musculoskeletal and Rheumatic diseases, National Institute of Rehabilitation, Mexico city, Mexico

**Keywords:** Inflammatory bowel disease, Spondyloarthritis, Ultrasound, Enthesis, Joint

## Abstract

**Background:**

Inflammatory bowel diseases are associated with a variety of extra-intestinal manifestations. The most frequent of these is joint involvement, which affects 16–33 % of IBD patients. Our aim was to evaluate the ultrasound prevalence of sub-clinical joint and entheseal involvement in patients with IBD without musculoskeletal symptoms, and to correlate the US findings with clinical and laboratory variables.

**Methods:**

We recorded the clinical and laboratory data of 76 patients with IBD, 20 patients with spondyloarthritis (SpA) and 45 healthy controls at three rheumatology centers. All of the IBD patients and healthy controls were clinically examined by a rheumatologist in order to confirm the absence of musculoskeletal symptoms, and all of the subjects underwent grey-scale (GS) and power Doppler (PD) US examinations of the second and third metacarpophalangeal joints, knees and lower limbs in order to detect joint or entheseal abnormalities.

**Results:**

A total of 1410 entheseal sites and 1410 joints were evaluated by US. Of the 76 patients with IBD, 64 (84.1 %) had at least one GS entheseal abnormality, and 11 (13.9 %) had more than one PD-positive entheseal site; 32 (42.1 %) showed sub-clinical joint involvement.

There was a significant difference between the IBD patients and healthy controls in terms of global entheseal, PD-positive entheseal, and joint involvement (*p* < 0.0001), but no difference between the IBD and SpA patients. Anti-neutrophil cytoplasmic antibodies predicted entheseal involvement in patients with IBD (OR 6.031; *p* = 0.015).

**Conclusions:**

The prevalence of sub-clinical joint and entheseal involvement was higher in IBD patients than healthy controls, but there was no difference between the IBD and SpA patients.

## Background

Nearly four million people worldwide are affected by inflammatory bowel diseases (IBD) [1], which are associated with a variety of extra-intestinal manifestations [2]. The most frequent of these is joint involvement, which affects 16–33 % of IBD patients, [2–4] whereas 5–10 % are affected by enthesitis [5].

Musculoskeletal involvement is a major concern in IBD patients as it increases disability and worsens their quality of life. However, its prevalence is frequently underestimated because of the transient manifestation of some oligoarticular patterns or the use of chronic corticosteroid treatment [6], because enthesitis is mistakenly attributed to overuse, or because the recognition of joint and tendon involvement may be delayed by the fact that gastroenterologists may not specifically enquire about musculoskeletal symptoms in everyday clinical practice [7]. It is therefore necessary to adopt a multidisciplinary approach in order to ensure that it is detected and appropriately treated early enough to avoid poor outcomes.

Ultrasonography (US) is a valid tool of detecting joint and tendon involvement early in patients with various rheumatic conditions. It is more sensitive than a clinical examination in revealing synovitis [8–13] and enthesitis [14–18], and can detect pathological changes even in the absence of symptoms [19–24].

The aims of this US study were to evaluate the prevalence of sub-clinical joint and entheseal involvement in patients with IBD, and correlate the findings with clinical and laboratory data.

## Methods

Patients with a definite diagnosis of IBD made by an experienced gastroenterologist on the basis of clinical, histological, endoscopic, radiological and laboratory data, and without any musculoskeletal symptoms were recruited by the outpatient gastroenterology and rheumatology departments of the study centers. There were two control groups: one consisting of patients with spondyloarthritis (SpA) diagnosed on the basis of the Assessment of SpondyloArthritis international Society (ASAS) criteria [25–27], and the other of age- and gender-matched healthy subjects.

All of the subjects underwent a rheumatological examination by an expert rheumatologist blinded to their clinical condition in order to assess musculoskeletal involvement, and all of the patients underwent an US examination of the second and third metacarpophalangeal (MCP) joints, knees and lower limbs. The clinical and US assessments were made separately one immediately after the other by two investigators who were unaware of the other’s findings.

The exclusion criteria were an age of <18 years, a body mass index (BMI) of >30, a history of any inflammatory, microcrystalline, degenerative or infectious musculoskeletal disease, lower limb peripheral neuropathy, a history of severe trauma, knee or ankle surgery, or corticosteroid injections in the examined structures. IBD patients with signs of synovitis or enthesitis at clinical examination were also excluded.

All subjects gave informed consent to participate in the study, which was conducted in accordance with the Declaration of Helsinki and approved by Comitato Etico dell’Azienda Sanitaria Unica Regionale di Ancona.

### Clinical assessment

Disease activity in all of the IBD patients was evaluated by a gastroenterologist using the Crohn’s Disease Activity Index (CDAI) [28] and a modified version of the ulcerative colitis (UC) Mayo Index [29] without the endoscopic assessment. CD remission was defined as a CDAI score of <150 score and UC remission as ≤3 bowel movements without blood.

Data concerning disease duration, the number of flares, anti-*Saccharomyces cerevisiae* (ASCA) and *perinuclear anti-neutrophil antibodies* (pANCA) profiles, HLA-B 27 status, the erythrocyte sedimentation rate (ESR), C-reactive protein (CRP) levels, current medications and BMI were recorded upon study entry.

The rheumatological evaluation was made by an experienced rheumatologist using a standard protocol that included confirmation of the absence of musculoskeletal symptoms or a history of any musculoskeletal disease, or any disorder that may have had musculoskeletal effects; the recording of all drugs received in the 12 weeks preceding study inclusion; a history of bone fracture and joint surgery; and a physical examination assessing the swelling and tenderness of the second and third MCP joints, knees, ankles, and first metatarsophalangeal (MTP) joint and entheses.

### US assessment

The US examinations were performed by one investigator in each study centre who was blinded to the subject status and to whom the subjects were asked not to mention their condition. Before beginning the study, the investigators had reached a consensus concerning on the scanning technique and the findings to search for. The machines used were a MyLab Twice (Esaote S.p.A., Genova, Italy), a MyLab 70XVG (Esaote S.p.A., Genova, Italy.) and a GE LOGIQ P5 (General Electric Company, Little Chalfont, United Kingdom), all of which were equipped with a 6–18 MHz broadband linear transducer. Each anatomical area (the second and third MCP joints, knees, ankles, and first MTP joints) was bilaterally scanned first in grey scale (GS) in order to detect any morphostructural changes, and subsequently with power Doppler (PD with a pulse repetition frequency of 500–1000 Hz and a Doppler frequency of 6–9.1 MHz) in order to detect abnormal blood flow. The examination also investigated the quadriceps, distal and proximal patellar, Achilles, and proximal plantar aponeurosis entheseal insertion.

During the multiplanar exploration of the MCP joints, the subject sat with his or her hands on the examination table and fingers extended but relaxed. The knee was scanned with the subject in supine decubitus with the knee in a neutral position, and the tibiotalar joint with the subject in supine decubitus, the knee flexed 45°, and the foot resting over its plantar aspect with slight plantar flexion. The MTP joints were scanned with the patient in supine decubitus, the knee flexed 45°, the foot resting over its plantar aspect, and the toes extended and relaxed [30]. During the examinations of the superior (quadriceps enthesis) and inferior pole of the patella (patellar enthesis), and the patellar enthesis at the anterior tibial tuberosity, the subject was supine with extended lower limbs; the Achilles tendon and proximal plantar aponeurosis were examined with the patient lying prone with feet hanging over the edge of the examination table at 90° of flexion [30].

All of the US findings indicative of enthesopathy were based on the OMERACT definition [31]. A PD abnormality was considered positive if at least a single vessel was present at < 2 mm of cortical bone and not at tendon body or bursal level.

The supra-patellar pouch, MCP, knee, ankle and MTP joints were assessed for synovial fluid and synovial hypertrophy. Synovial fluid was considered documented in the presence of anechoic or hypoechoic joint cavity widening displacable/compressible by ultrasound probe, and synovial proliferation in the presence of abnormal hypo- or hyperechoic not compressible material inside the joint cavity [31]. All sites were scanned in longitudinal and transversal views and the alterations considered present if seen in both views. Both the GS and PD findings were qualitatively assessed (i.e. present/absent).

### Statistical analysis

The continuous variables are expressed as mean values ± standard deviation (SD) or median values and interquartile range (IQR) as appropriate, and the categorical variables as percentages. The associations between joint/PDUS/entheseal abnormalities and clinical variables were evaluated using the Mann–Whitney test in the case of continuous variables and the χ^2^ test in the case of categorical variables. A binary logistic regression model containing all of the variable that were significant in the univariate analysis was used to establish the variables predicting joint/PDUS/entheseal abnormalities.

The data were analyzed using SPSS software, version 20 (Chicago, Illinois, 2011), and a value of *p* < 0.05 was considered statistically significant.

## Results

One hundred and forty one subjects were recruited: 76 with IBD (43 with CD, and 33 with UC), 20 patients with established SpA (12 Ankylosing spondylitis, 8 non-radiographic axial SpA), and 45 healthy controls. Table 1 shows the characteristics of the IBD patients.

**Table 1:** Clinical and laboratory characteristics of IBD patients

Crohn’s disease/Ulcerative colitis	43/33 patients
Age	39.93 ± 14.02 years
BMI	23.36 ± 4.13 kg/m^2^
Disease duration	7.91 ± 6.18 years
Active/inactive bowel disease	51.3 %/48.7 %
Disease flares	2.53 ± 1.97
ASCA positive	25 % (CD)
pANCA positive	18.4 % (17 % UC + 1.4 % CD)
HLA B 27 positive	2.6 %
CRP	0.69 ± 0.96 mg/dL
ESR	14.52 ± 11.48 mm/h
Medications	
- Corticosteroids	19.7 %
- 5 ASA	42.1 %
- Anti-TNFα	32.9 %
- Azathioprine	28.9 %
- Cyclosporine	1.3 %

**Fig. 1:**
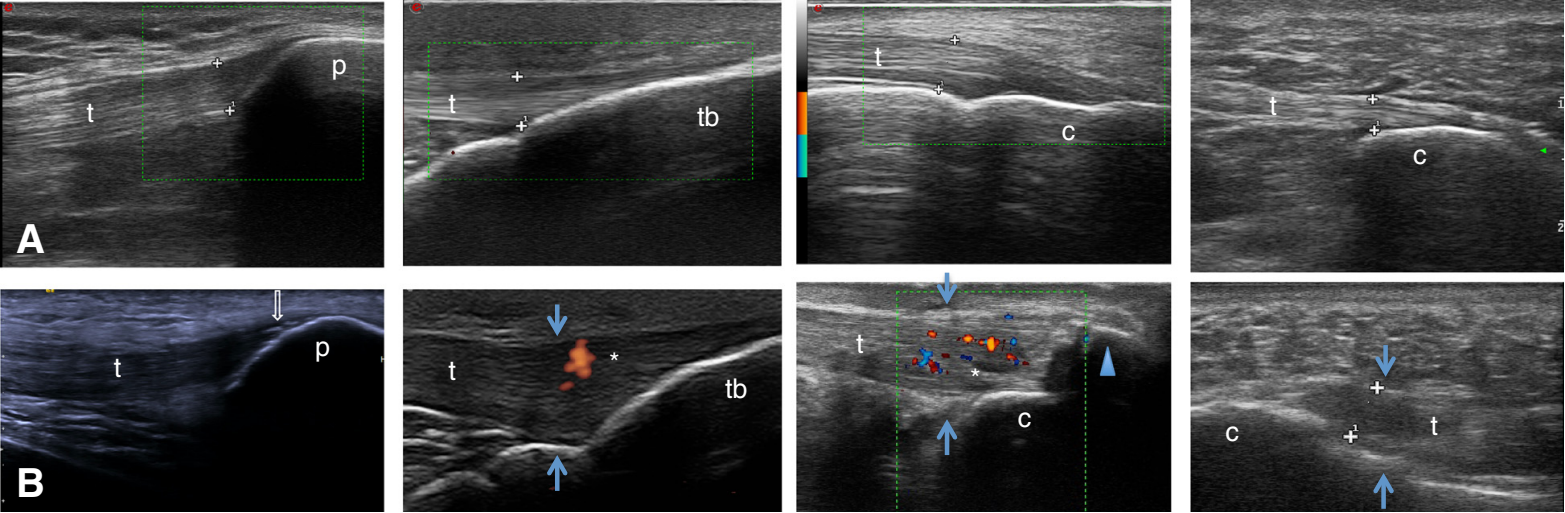
Upper panels: normal enthesis. A1) quadriceps; A2) distal patellar; A3) Achilles; A4: proximal plantar aponeurosis. Lower panels: pathological enthesis showing increased thickness (blue arrows), enthesophyte (blue arrowhead), calcifications (open arrow) and abnormal blood flow (asterisk). B1) quadriceps; B2) distal patellar; B3) Achilles; B4) proximal plantar aponeurosis. t – tendon; c – calcaneus bone; tb –tibial bone

US was used to evaluate a total of 1410 entheseal sites and 1410 joints. Of the 76 patients with IBD, 84.1 % had at least one entheseal abnormality (from a total of 60 per patient), 50 % had at least three, 40.8 % more than five, and 17.1 % had more than 10 abnormalities (GS and PD); 24 (31.6 %) had at least one PD-detected abnormality, and 11 were PD-positive at more than one entheseal site. The patellar tendon was the most affected entheseal site (a total of 218 detected abnormalities out of 1824), followed by the quadriceps tendon. Ultrasound normal and abnormal findings at the four entheseal sites are exhibited in Fig. 1. Thirty-two of the 76 IBD patients showed sub-clinical joint involvement. Excluding the first MTP (because of the frequency of paraphysiological abnormalities), the knee was the most affected joint (24 irregularities out of 456), followed by the ankle with eight.

No significant difference was found in the detection rates of abnormal findings in each institution.

Table 2 shows the prevalence of joint and entheseal involvement. There was a significant difference between the IBD patients and healthy controls (*p* < 0.0001), but no difference between the IBD and SpA patients, and no apparent difference between the patients with CD and UC (Table 3).

**Table 2:** Ultrasound findings by disease status

	IBD	SpA	HC
All joints	21 (48.8 %)	8 (40 %)	4 (8.9 %)*
MCP	3 (5.2 %)	1 (1.31 %)	0 (0 %)*
Knee	12 (15.8 %)	6 (30 %)	2 (4.4 %)*
Ankle	7 (11.3 %)	7 (35 %)**	0 (0 %)*
1^st^ MTP	17 (22.4 %)	8 (40 %)	2 (4.4 %)*
All entheseal sites	63 (82.9 %)	20 (100 %)	13 (28.9 %)*
Quadriceps tendon	31 (40.8 %)	11 (55 %)	2 (4.4 %)*
Patellar tendon	51 (67.1 %)	19 (95 %)**	2 (4.4 %)*
Achilles tendon	33 (43.4 %)	13 (65 %)	5 (11.1 %)*
Plantar fascia	19 (25 %)	12 (60 %)**	2 (4.4 %)*
Entheseal power Doppler	25 (32.9 %)	11 (55 %)	0 (0 %)*

Number of subjects with the alteration and their relative prevalence (%)

*IBD* inflammatory bowel disease, *SpA* spondyloarthritis, *HC* healthy controls

*IBD *vs* HC *p* < 0.0001; **IBD vs SpA *p* < 0.01

**Table 3:** Ultrasound findings in CD and UC

	CD	UC
All joints	11 (25.5 %)	10 (30.3 %)
MCP	3 (6.9 %)	0 (0 %)
Knee	4 (9.3 %)	8 (24.2 %)
Ankle	4 (9.3 %)	3 (9.1 %)
1^st^ MTP	11 (25.5 %)	6 (18.2 %)
All entheseal sites	37 (86 %)	26 (78.8 %)
Quadriceps tendon	16 (37.2 %)	15 (45.5 %)
Patellar tendon	31 (72.1 %)	20 (60.6 %)
Achilles tendon	16 (37.2 %)	17 (51.5 %)
Plantar fascia	8 (18.6 %)	11 (33.3 %)
Entheseal power Doppler	15 (34.9 %)	10 (30.3 %)

Number of subjects with the alteration and their relative prevalence (%)

No significative difference was found between groups

*CD* Chron disease, *UC* Ulcerative colitis

There was no association between joint involvement and the clinical or laboratory data of the IBD patients but, interestingly, global US entheseal abnormalities were associated with the presence of pANCA (*p* = 0.011) and current treatment with azathioprine (*p* = 0.014). pANCA also predicted entheseal involvement in the IBD patients (OR 6.031; *p* = 0.015). PD-detected entheseal abnormalities correlated with the number of IBD flares (*p* = 0.009), CRP (*p* = 0.044) and current treatment with azathioprine (*p* = 0.005).

No other associations were found between the clinical and laboratory data and US-detected entheseal involvement (Tables 4, 5 and 6).

**Table 4:** Clinical and laboratory data by PD- and GS-detected entheseal abnormalities

	PD-detected entheseal abnormalities			GS-detected entheseal abnormalities			GS + PD detected entheseal abnormalities		
	present	absent	*p*	present	absent	*p*	present	absent	*p*
Age^a^	52 (23.5-58.3)	38 (29–45)	0.707	40 (28–56)	33.5 (24.5-38.5)	0.155	40 (28–56)	33.5 (24.5-38.5)	0.102
BMI^a^	22.59 (19.9-24.6)	23.24 (19.9-25.9)	0.608	23.4 (20.9-26.4)	21.9 (19.7-24.4)	0.297	23.24 (20.9-26.4)	21.87 (19.7-24.4)	0.297
Disease duration^a^	6 (2.3-9.5)	7 (4.5-12)	0.695	7 (4–12)	6.5 (4.5-10)	0.724	7 (4–12)	6.5 (4.5-10)	0.972
Disease flare^a^	2.50 (2–3.75)	3 (2–5)	0.009*	4 (2–5)	2 (1.75-53)	0.204	4 (2–5)	2 (1.75-3)	0.295
Active disease^b^	21	79	0.883	0	100	0.53	9.1	91.9	0.721
ESR^a^	8.5 (5.3-9.8)	10 (6–17.5)	0.224	8 (5–11)	14.5 (10–29)	0.116	8 (5–11)	14.5 (10–29)	0.196
CRP^a^	0.35 (0.19-075)	0.19 (0.09-0.8)	0.044*	0.19 (0.1-0.61)	0.4 (0.1-1.3)	0.116	0.19 (0.1-0.6)	0.4 (0.1-1.35)	0.796
ASCA^b^	15.8	84.2	0.127	78.9	21.1	0.991	30	70	0.822
pANCA^b^	21.4	78.6	0.476	57.1	42.9	0.022*	16	84	0.011*
Steroids^b^	14.3	85.7	0.271	14.2	85.8	0.835	9.4	90.6	0.909
Anti-TNFα^b^	40	60	0.356	92	8	0.14	37.5	62.5	0.05
AZA^b^	9	91	0.005*	68.1	31.9	0.030*	23.4	76.6	0.014*
5 ASA^b^	40	60	0.745	41.2	58.8	0.745	39.1	60.0	0.727

^a^median values (interquartile ranges), Mann–Whitney U test; ^b^percentages (%), χ^2^ test

*PD* power Doppler, *GS* grey scale, *CRP C*-reactive protein, *ESR* erythrocyte sedimentation rate, *pANCA* anti-neutrophil cytoplasmic antibodies, *ASCA* anti-*Saccharomyces cerevisiae* antibodies, *AZA* azathioprine, 5 *ASA* 5 asa compounds; *p < 0.05

**Table 5:** Univariate analysis: odds ratios

	PD-detected entheseal involvement			PD + GS-detected entheseal involvement			GS-detected entheseal involvement		
	Odds Ratio	95 % CI		Odds Ratio	95 % CI		Odds Ratio	95 % CI	
		Lower	Upper		Lower	Upper		Lower	Upper
Azathioprine	0.268	0.078	0.921	0.135	0.029	0.635	0.219	0.060	0.790
pANCA	0.228	0.06	0.859	-			0.190	0.049	0.743

*GS* grey scale, *PD* power Doppler, *pANCA*: anti-neutrophil cytoplasmic antibodies, *CI* confidence interval

**Table 6:** Multivariate logistic regression models predicting GS- and PD-detected entheseal abnormalities

	GS-detected entheseal abnormalities				PD-detected entheseal abnormalities				GS + PD-detected entheseal abnormalities			
	Exp (B)	95 % IC		*p*	Exp (B)	95 % CI		*p*	Exp (B)	95 % CI		*p*
		Lower	Upper			Lower	Upper			Lower	Upper	
Azathioprine	2.915	0.775	10.971	0.114	4.210	0.797	22.252	0.091	3.831	0.934	15.716	0.062
pANCA	4.755	1.195	18.915	0.027	-				6.031	1.412	25.757	0.015
Disease flares	-				0.704	0.486	1.019	0.063	-			
CRP	-				1.128	0.641	1.985	0.677	-			

*GS* grey scale, *PD* power Doppler, *pANCA* anti-neutrophil cytoplasmic antibodies, *CI* confidence interval, *CRP* C-reactive protein

## Discussion

This is the first multicenter study to explore joint and entheseal involvement in IBD patients with no signs or symptoms of musculoskeletal disease and, to the best of our knowledge, the first time that this has been compared in IBD and SpA patients.

Our findings indicate that the prevalence of sub-clinical entheseal and joint involvement is in high with patients with IBD. The prevalence of sub-clinical entheseal involvement is in line with the 92.6 % reported by Bandinelli et al. [23], although they found a higher prevalence of PD-detected involvement (32.9 % *vs* 16 %). Enthesitis is often the first sign of SpA in young adults [32], but only a small percentage of cases are discovered by means of a clinical examination: for example, in a cross sectional study of 130 Brazilian IBD patients, a clinical assessment identified enthesitis in only (5,4 %) [2], and Balint found the sensitivity of a clinical examination was low in patients with SpA [14].

As found in previous studies [23], the most affected entheseal site in our patients was the patellar tendon, followed by the Achilles tendon, which is often indicated as being the most affected in patients with SpA [33, 34]. On the contrary, the prevalence of frequently described plantar fascia abnormalities was lowest, possibly because the thicker layer of subcutaneous tissue and skin in this anatomical region reduces the sensitivity of US.

Arthritis is a common finding, and has been reported in 17–39 % of patients with IBD [6]. We found that the prevalence of joint alterations was as high as 48.8 %, although one magnetic resonance imaging (MRI) study of 11 IBD patients with painful hand joints without clinical synovitis and 11 without painful joints found no signs of synovitis in the latter [35]. However, the number of patients was small and the study only considered hand joints.

Some studies have confirmed the relationship between sub-clinical gut and joint inflammation [32, 36–38]. The GIANT cohort study found microscopic gut inflammation in 46 % of the SpA patients (the prevalence was higher in those with axial SpA than those with peripheral SpA), and one recent MRI study [39] found that chronic gut inflammation was associated with sacroiliac joint bone marrow edema.

In our study we found that azathioprine was associated with increased entheseal involvement in IBD patients. It has been shown a direct relationship between the extent of colonic disease and the presence of extraintestinal manifestations [4]. Patients under treatment with azathioprine could have higher bowel disease activity and could also have more extraintestinal features including entheseal involvement. It is also known that azathioprine is not an effective pharmacological treatment in SpA and therefore these patients may have more entheseal manifestations.

We also found an association between pANCA and entheseal involvement in IBD patients. It has been suggested that atypical pANCA cross-react with luminal bacteria and the environmental antigens driving the inflammatory process [40], and they have also been found to be associated with isolated colonic disease [41, 42] and 4-fold risk of undergoing azathioprine treatment [41]. Remarkably, colectomy seems to protect IBD patients against the development of peripheral arthritis [43].

Interestingly, we did not find any difference in US-revealed entheseal involvement between our patients with IBD and those with SpA. A multidisciplinary approach is useful when following up IBD patients because the transient nature of the oligoarticular pattern of arthritis, the use of corticosteroids to manage IBD flares [6] or the attribution enthesitis to overuse, may lead clinicians to underestimate the prevalence of arthritic involvement. Moreover, gastroenterologists may not specifically ask about musculoskeletal symptoms in clinical daily practice, thus leading to the delayed recognition of joint and entheseal involvement [7].

This study has some limitations. First of all, as US is sensitive to small alterations, the fact that the data were recorded dichotomously may have led to an overestimate of particularly joint involvement. Secondly, the prevalence of pANCA was lower in our patients than that reported in the literature [40–42], and this may have affected our findings concerning their predictive value for entheseal involvement. Thirdly, the small study cohort did not allow us to measure the real dimension of the prevalence of entheseal involvement in IBD patients. Fourthly, the study is not powered enough to detect differences between IBD and SpA groups. We estimate a sample size of 49 for entheseal involvement and 500 for articular involvement considering a type I error of 0.05 and power of 80 %.

## Conclusion

We found a high prevalence of sub-clinical joint inflammation in IBD patients without any musculoskeletal signs or symptoms, and an association between pANCA and entheseal involvement, but further studies are necessary to confirm the latter finding. However, the question as to how such patients should be treated and followed up remains open as prospective studies are necessary in order to clarify the prognostic value of our findings.

## Abbreviations

IBD: inflammatory bowel disease
CDAI: Crohn’s Disease Activity Index
SpA: spondyloarthritis
HC: healthy controls
CD: chron disease
UC: ulcerative colitis
PD: power Doppler
GS: grey scale
CRP: c-reactive protein
ESR: erythrocyte sedimentation rate
pANCA: anti-neutrophil cytoplasmic antibodies
ASCA: anti-*Saccharomyces cerevisiae* antibodies
AZA: azathioprine
5 ASA: 5 asa compounds
