# Erratum to: Hidden musculoskeletal involvement in inflammatory bowel disease: a multicenter ultrasound study

**DOI:** 10.1186/s12891-016-1098-4

**Published:** 2016-06-01

**Authors:** João Rovisco, Cátia Duarte, Alberto Batticciotto, Piercarlo Sarzi-Puttini, Antonella Draghessi, Francisco Portela, Marwin Gutierrez

**Affiliations:** Rheumatology Department, Centro Hospitalar e Universitário de Coimbra, Praceta Prof Mota Pinto, Coimbra, 3000-075 Portugal; Rheumatology Unit, L. Sacco University Hospital, Milan, Italy; Clinica Reumatologica, Università Politecnica delle Marche, Jesi, Ancona Italy; Gastroenterology Department, Centro Hospitalar e Universitário de Coimbra, Coimbra, Portugal; Division of Musculoskeletal and Rheumatic diseases, National Institute of Rehabilitation, Mexico city, Mexico

## Erratum

After publication of the original article [1], it came to the authors’ attention that there were spelling errors in the surnames of two of the authors. The surnames of A. Batticciotto and A. Draghessi were misspelled but appear correctly in this erratum.
